# *Streptococcus suis* Meningitis and Bacteremia in Man, French Guiana

**DOI:** 10.3201/eid1909.121872

**Published:** 2013-09

**Authors:** Magalie Demar, Carine Belzunce, Christine Simonnet, Alain Renaux, Philippe Abboud, Antoine Okandze, Corrine Marois-Créhan, Felix Djossou

**Affiliations:** Centre Hospitalier de Cayenne, Cayenne, French Guiana (M. Demar, C. Belzunce, C. Simonnet, A. Renaux, P. Abboud, A. Okandze, F. Djossou);; Agence Nationale de Sécurité Sanitaire, Ploufragan, France (C. Marois-Créhan)

**Keywords:** Streptococcus suis, bacteria, meningitis, bacteremia, human infection, French Guiana

**To the Editor:** *Streptococcus suis* is a major swine pathogen and is increasingly recognized as the cause of an emerging zoonosis in humans. It is responsible for severe systemic infections, most commonly meningitis and sepsis, which lead to high rates of illness and death. Serotype 2 is considered to be the strain most pathogenic for humans and pigs (*1*,*2*).

The first reported infection in a human was in Denmark (*3*), but human infections have been now described worldwide, with a predominance in countries in Southeast Asia (*1*,*2*,*4*). However, only 2 cases of human infection have been described in South America, in Argentina in 2005 (*5*) and 2008 (*6*). One infection was related to a serotype 2 strain (*5*). We report a human case of *S. suis* infection with meningitis and bacteremia in an immunocompetent patient living in French Guiana.

In August 2011, a 42-year-old Haitian man was admitted to Cayenne Hospital (Cayenne, French Guiana) because of a 3-day history of fever, headache, vertigo, and vomiting. He was an unemployed illegal immigrant who had lived in French Guiana for 4 years. He had no particular medical history and no risk factors for immunodeficiency. At admission, he was lethargic, weak, and frail. He had a stiff neck, vestibular dysfunction, ataxia, and nystagmus. He did not have hearing loss or skin abnormalities. Cranial computed tomography with contrast and gadolinium-enhanced cranial magnetic resonance imaging did not show any abnormal findings.

Blood tests indicated thrombocytopenia (106 × 10^9^ platelets/L), lymphopenia (0.57 ×10^9^ lymphocytes/L), a high level of C-reactive protein (170 mg/L) and mild cytolysis (3.8-fold increase in aspartate aminotransferase and 2.5-fold increase in alanine aminotransferase levels). There was no hyperleukocytosis (leukocyte count 7.7 × 10^9^ cells/L). Cerebrospinal fluid (CSF) was slightly opalescent and had a protein level of 1.16 g/L, a glucose level of 2.2 mmol/L, and 270 leukocytes/μL (79% neutrophils and 21% lymphocytes). Gram staining of CSF showed numerous gram-positive cocci in pairs and short chains.

CSF and blood cultures after 12 and 48 hours, respectively, of incubation showed numerous alpha-hemolytic colonies on blood and chocolate agar. The bacteria were gram positive, optochin resistant, and catalase negative, and were reliably identified by using API 20 Strep (bioMérieux, Marcy l’Etoile, France) as *S. suis* (probability 99.7%).

Antimicrobial drug susceptibility tests were conducted by using Mueller-Hinton agar and the disk diffusion method. An E-test was performed for ampicillin (MIC<0.016 μg/mL). The isolate was resistant to gentamicin (500 μg/L), tetracycline, and norfloxacin, and sensitive to penicillin, ampicillin, amoxicillin, oxacillin, cefotaxim, pristinamycin, vancomycin, teicoplanin, trimethoprim/sulfamethoxazole, erythromycin, and lincomycin in comparison with published data (*7*). Slide agglutination with type-specific hyperimmune serum and specific multiplex PCR (*8*,*9*) identified the isolate as serotype 2. Multilocus sequence typing identified the isolate as a member of the sequence type 1 complex, which has spread throughout most countries in Europe. This complex has been strongly associated with isolates from patients with septicemia, meningitis, and arthritis (*10*).

After *S. suis* was identified, the patient reported contact with swine and that he had slaughtered 4 pigs 24 hours before onset of symptoms. During this activity, he injured his left thumb. The patient was empirically given a 2-day course of intravenous, high-dose ceftriaxone (3 g, 2×/d), which was replaced by intravenous high-dose amoxicillin (3g, 4×/d) for 14 days.

While the patient was receiving treatment for 2 days, moderate bilateral hypoacousia developed, which required adjunctive corticoid therapy. The hypoacousia developed into severe hearing loss in the left ear. Audiograms showed moderate sensorial hearing loss (50 dB) in the right ear on day 7 of treatment, which resolved 1.5 years later, and complete hearing loss in the left ear on day 7 of treatment, which that gradually decreased. However, the patient still has severe sensorinoral hearing loss (80 dB) in the left ear. The patient did not have signs or symptoms of endocarditis by cardiac ultrasonography.

Little data are available for circulation and epidemiology of *S. suis* in South America (*3*). We report a human case of *S. suis* infection with meningitis and bacteremia. Clinical and laboratory data, microbiological findings, and outcome for this case-patient were similar to those of the 2 case-patients reported in Asia (*1*,*2*).

Our study shows that in nonendemic areas, infection with this pathogen, although not frequently reported, should be considered when diagnoses are made for patients who work in piggeries. Persons with occupational exposure to swine or pork products should be educated and made aware of this risk for infection.
